# Seroprevalence of brucellosis in sheep and goats in the Arabian Gulf region

**DOI:** 10.14202/vetworld.2020.1495-1509

**Published:** 2020-08-06

**Authors:** M. Ebid, A. El Mola, F. Salib

**Affiliations:** 1 Department of Animal Care and Medicine, General Organization of Veterinary Service, Giza, Egypt; 2 Department of Medicine and Infectious Diseases, Faculty of Veterinary Medicine, Cairo University, Egypt

**Keywords:** Arabian gulf, *Brucellosis*, goats, indirect enzyme-linked immunosorbent assay, polymerase chain reaction, Rose Bengal, seroprevalence, sheep

## Abstract

**Background and Aim:**

Brucellosis is a zoonosis that occurs worldwide. There were more efforts to control brucellosis in all countries. This study was performed to determine the seroprevalence of brucellosis in sheep and goats in some areas in the Arabian Gulf.

**Materials and Methods:**

The study analyzed 8500 sera from non-vaccinated sheep and goats. Animals included 6441 sheep (3420 from farms and 3021 from quarantine) and 2059 goats (1580 from farms and 479 from quarantine). Sera were tested using the Rose Bengal Plate Test (RBPT) and confirmed with an indirect enzyme-linked immunosorbent assay (i-ELISA) test. Final confirmation analyzed blood samples from confirmed infected animals (n=30, 23 sheep and seven goats) using polymerase chain reaction (PCR) and culture.

**Results:**

The serological examination showed that 62/8500 of animals (0.729%, confidence interval [CI] 95% 0.57-0.94) were seropositive for brucellosis. Thirteen of 5000 (0.26%, CI 95% 0.15-0.45) and 49/3500 (1.4%, CI 95% 1.1-1.8) of animals from farms and quarantine were seropositive, respectively. Out of the 6441, 46 (0.71%) sheep and 16/2059 (0.78%) goats were seropositive. i-ELISA confirmed 41/62 RBPT-positive animals – 41/8500 (0.482%, CI 95% 0.36-0.65). Eight of 5000 of these animals (0.16%, CI 95% 0.08-0.32) and 33/3500 (0.94%, CI 95% 0.67-1.3) were confirmed positive in farms and quarantine, respectively. Thirty of 6441 (0.466%) and 11/2059 (0.534%) cases were positive in sheep and goats, respectively. PCR confirmed 18 of 41 positive animals (0.212% of all sera, CI 95% 0.13-0.34) identified by both RBPT and i-ELISA. Three of 5000 (0.06%, CI 95% 0.019-0.19) and 15/3500 (0.429%, CI 95% 0.26-0.71) from farms and quarantine were confirmed positive. Tissue samples (uterine, supra-mammary, testicular, and accessory glands lymph node) were collected from positive animals, as detected by RBPT and i-ELISA, at culling or slaughtering. Using *in vitro* culture, 14/30 were confirmed positive – 3/7 from farms (two sheep and one goat) and 11/23 from quarantine (nine sheep and two goats). Biovar 1 was dominant. PCR confirmed 23/30 tissue samples, 4/7 from farms (three sheep and one goat), and 19/23 from quarantine (15 sheep and four goats).

**Conclusion:**

The overall brucellosis rate in sheep and goats is 0.48%, with fewer animals from farms testing positive (0.16%) in this area of the Arabian Gulf. The infection appears to be well controlled, and continuous effort is still needed to maintain control and completely eradicate brucellosis. Additional support is needed for testing and slaughterhouse monitoring. In quarantine (imported animals), brucellosis infection in the slaughterhouse (0.94%) could pose a risk for transmission and spread of infection. The effort is needed to monitor this threat, and PCR is a sensitive and time-saving test for brucellosis diagnosis. All 14 confirmed positive samples were Biovar 1 dominant.

## Introduction

*Brucella melitensis*, in particular, is a reemerging pathogen in the Mediterranean, Arabian Gulf, and Middle East regions. The pathogen causes severe disease in livestock and has an enormous impact on the economy of developing countries [1]. Infection of sheep and goats causes abortions, weak offspring, reduced milk production, weight loss, infertility, and lameness. The disease has been eradicated in some countries, though the cost of surveillance to maintain a *B. melitensis*-free state remains high. The pathogen displays three Biovars (1, 2, and 3). Brucellosis is a common disease and an important zoonosis in the Mediterranean area. Continuous progress is notable for brucellosis control, yet it is still a significant public health hazard and of great economic importance [2-4]. Various methods of control have been adopted in different countries based on the elimination of infected animals detected by serological and other diagnostic tests, other control methods based on vaccination [5]. One of the most common zoonotic diseases has economic importance worldwide and has significant public health is Brucellosis [6,7]. Brucellosis is caused by a Gram-negative bacterium in the genus *Brucella*. These bacteria are facultatively anaerobic, non-motile, and intracellular coccobacilli. Brucellosis affects a wide range of mammals, including man, sheep, camels, cattle, goats, swine, and wildlife [1,8-10].

All three biovars cause disease in small ruminants, but their geographic distribution varies. Biovar 1 is most common in Libya and Oman, and Biovar 2 is dominant in Turkey and Saudi Arabia. Biovar 3 is most commonly encountered in Egypt, Jordan, and Tunisia. Saudi Arabia, Iran, the Palestinian Authority, Syria, Jordan, and Oman show the highest incidence rates for human brucellosis. Bahrain is reported as free of *B. melitensis* [11,12] Typically, Rose Bengal Plate Test (RBPT) is used for field screening for brucellosis, sometimes along with indirect enzyme-linked immunosorbent assay (i-ELISA) [12-16]. Better approaches used more than one serological test, accompanied by molecular detection and culture, for the best diagnosis and control [11,17]. Nucleic acid amplification methods, such as polymerase chain reaction (PCR), are rapid, sensitive, and highly specific and can counteract limitations of conventional detection methods [18,19]. Furthermore, some bacterial infections, such as *Chlamydia abortus*, interfere with brucellosis in small ruminants [20]. A new PCR method shows high specificity and sensitivity for brucellosis diagnosis [21,22]. The technique can also be used to detect brucellosis in milk and milk products [23,24].

This study was conceived to address the lack of studies on the prevalence of brucellosis in sheep and goats in the gulf area and to update data on infection rate. Especially, the study aims to assess a wide range of incidence previously reported and to evaluate control methods, reporting by local authorities, and veterinary resources. The present study focused specifically on seroprevalence of brucellosis in sheep and goats in some areas surrounding the Arabian Gulf.

## Materials and Methods

### Ethical approval

This study was approved by the Research Ethics Committee at Cairo University. All samples, including blood samples and tissue samples, were collected following standard procedures without any animal harm with the acceptance of owners and local veterinary authority in Gulf Cooperation Council area.

### Study area and study period

The study was performed in the Arabian Gulf region, characterized by a desert climate−scorching in summer and mild in winter. Only two main seasons exist; a hot period from April to October and a cooler period from December to February. Within the hot season, extreme heat extends from May to mid-October. March and November are intermediate warm months [25]. The present cross-sectional study was conducted between September 2014 and May 2018 with sheep and goat from farms, quarantine, and abattoirs in the Arabian Gulf region.

### Study population

The study involved 85,000 non-vaccinated small ruminants and 855 farms represented by 8500 animals. The samples were collected animals from unorganized farms in four territories plus quarantine and eight organized farms. In selected localities, smaller administrative units or farms, as well as sheep flocks, and individual animals, were randomly selected and sampled using a simple random method [26].

**Figure-1:**
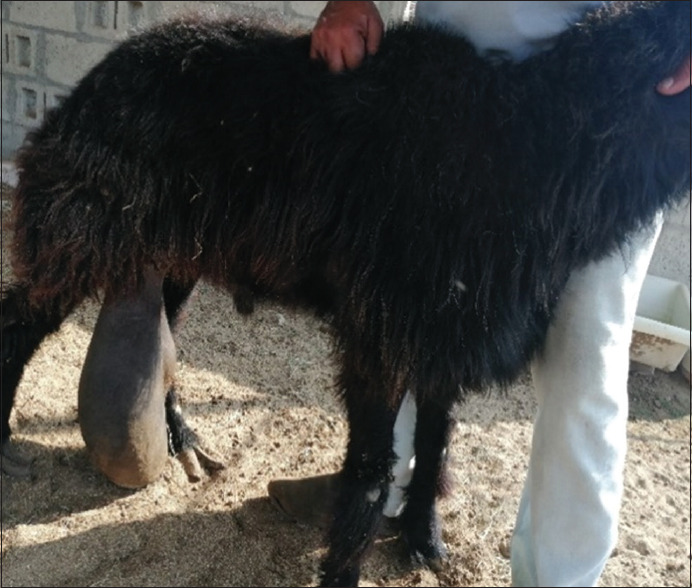
Unilateral orchitis in ram.

**Figure-2:**
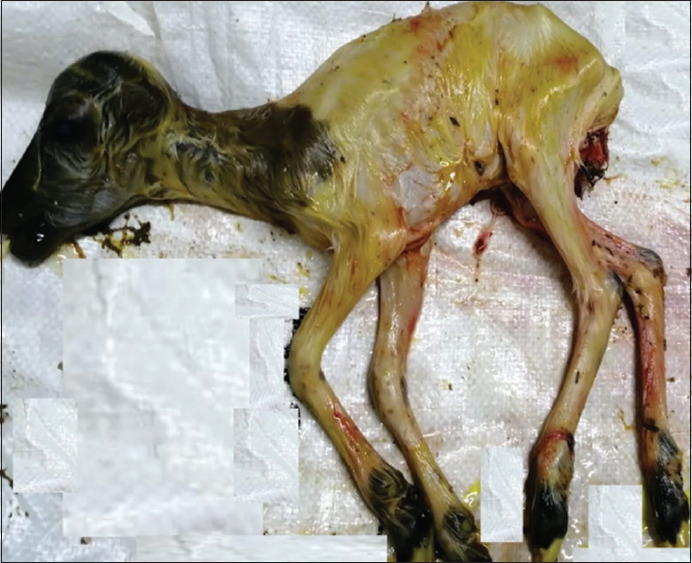
Aborted fetus of sheep.

Test animals included 6441 sheep and 2059 goats. Group 1 includes Four thousand animals from 252 unorganized farms in four territories, and 1000 animals from eight organized farms, (3420 sheep and 1580 goats). Group 2 includes 3500 animals from quarantine after import from different regions and countries (3021 sheep and 479 goats). The random animal selection was applied for farms and for the animals at farms. However, when farms noted gynecological problems, the samples were collected from both sick and healthy animals – full information about each animal – breed, sex, vaccination, and pathogenic condition was recorded. Images of animal conditions, including orchitis, abortion, mastitis, endometritis, and retained placenta, were taken. Figures-1 and 2 are examples of pathological conditions observed in the field.

### Sample size

The sample size (n) needed to assess the prevalence of brucellosis, 8500, was calculated using a standard formula [26,27]. The sample distribution between localities was 41.2% (n=3500), samples were from quarantine, 47% (n=4000) from unorganized farms, and 11.8% (n=1000) from organized farms. Animals were selected from unorganized farms in four territories, the Northern Territory, 17.6% (n=1500), Southern, 14.1% (n=1200), Moharaq, 2.4% (n=200), and Middle, 12.9% (n=1100). Data are summarized in Table-1. The sample size calculation used a confidence level of 95% and an error margin of 1%.

**Table-1:** Sample collected from animals in farms and quarantine.

Group	Type	Territory	Number of animals	Number of farms	Number of samples	Percentage of sample	Number of farms	Number of sheep samples	Number of farms	Number of goat samples	Number of farms
Group 1 farms	Unorganized farms	Northern terr.	12000	356	1500	17.6	85	789	74	711	52
		Southern terr.	28000	236	1200	14.1	72	912	69	288	27
		Moharaq terr.	3000	73	200	2.4	32	148	19	52	5
		Middle terr.	7000	182	1100	12.9	62	688	75	412	39
	Organized farms		5000	8	1000	11.8%	8	883	8	117	5
	Total (Group 1) farms		55000	855	5000	58.8%	259	3420	245	1580	128
Group 2	Quarantine		30000	0	3500	41.2%	0	3021	0	479	0
Total			85000	855	8500	100%	259	6441	238	2059	124

### Sample collection

#### Blood samples

Whole blood was collected from the jugular vein of each animal using two sterile Vacutainer tubes (10 ml of each). One tube without anticoagulant was used for serological tests, and the second tube with anticoagulant (EDTA) was labeled with codes describing animal and herd data recorded on investigation forms. Blood samples were kept in an upright position for 30 min in a cool place and then centrifuged at 3000 rpm for 20 min. The serum was collected by a micropipette and placed in Eppendorf tubes. Serum samples and blood samples were kept at −20°C until used, as recommended by OIE [28]. Blood samples (Table-2) were collected for analysis by RBPT, i-ELISA, and PCR.

**Table-2:** Blood sample collected from animals.

Group	Type	Territory	Number of samples	Number of farms	Number of sheep samples	Number of farms	Number of goat samples	Number of farms
Group 1 farms	Unorganized farms	Northern terr.	1500	85	789	74	711	52
		Southern terr.	1200	72	912	69	288	27
		Moharaq terr.	200	32	148	19	52	5
		Middle terr.	1100	62	688	75	412	39
	Organized farms		1000	8	883	8	117	5
	Total (Group 1) farms		5000	259	3420	245	1580	128
Group 2	Quarantine		3500	0	3021	0	479	0
Total			8500	259	6441	238	2059	124

#### Tissue samples

Tissue specimens from *Brucella* seropositive animals after slaughter were collected for bacteriological and PCR examination. Tissue samples included uterus, uterine, and supra-mammary lymph nodes in females, testis, testicular lymph nodes, ampules, and epididymis, including surround fat. Some lymph nodes, especially retropharyngeal nodes, were also collected. Tissues were packed as resected in disposable packaging, transferred hygienically to the laboratory, and kept frozen at −20°C till cultured and tested, as recommended by OIE [28]. All samples collected from one animal considered as one sample. Tissues were collected from 30 animals (Table-3) for examined by bacterial culture and PCR.

**Table-3:** Tissue samples collected from confirmed positive reactors.

Group	Type	Territory	Total	Sheep tissue sample			Goats tissue sample		
	
				Sheep	Number of samples	Sample collected	Goats	Number of samples	Sample collected
Group 1	Unorganized farms	Nortdern terr.	2	1	1	Uterus, ovaries, and lymph nodes	1	1	Uterus, ovaries and lymph nodes
		Soutdern terr.	2	2	2	Uterus, ovaries, and lymph nodes	0	0	-----------------
		Moharaq terr.	0	0	0	-----------------------------	0	0	------------------
		Middle terr.	2	1	1	Uterus, ovaries, and lymph nodes	1	1	Uterus, ovaries and lymph nodes
	Organized farms		1	1	1	Uterus, ovaries, and lymph nodes	0	0	--------------
Group 2	Quarantine		23	18	1	Uterus, ovaries, and lymph nodes	5	2	Testis and its accessory glands and lymph node
					7	Testis and its accessory glands and lymph node		3	Accessory glands and lymph node (castrated male)
					10	Accessory glands and lymph node (castrated male)			
Total			30	23	23		7	7	

### Serological analysis of samples

#### RBPT

Serum samples were tested using the RBPT antigen, according to Alton *et al*. [29]. Briefly, 30 μL of serum and 30 μL of RBT antigen combined on the white ceramic plate and carefully mixed. The plate was agitated for 4 min, and the degree of agglutination was recorded as a grade from 0, +, ++, and +++ [29]. Grade (0) indicates the absence of agglutination, grade (+) indicate barely visible agglutination, grade (++) indicates finely dispersed agglutination, and grade (+++) indicates coarse clumping. The samples with grade (+, ++, and +++) were considered positive. Positive and negative control sera for comparison of the results were used.

#### i-ELISA

All samples that tested positive by RBPT were further analyzed by i-ELISA for confirmation. i-ELISA was performed following ELISA kit manufacturer’s instructions “(ID. vet, ID screen, Brucellosis serum indirect multispecies, rue Louis Pasteur-Grabels – France).”

#### PCR

All positive samples that tested positive in both RBPT and i-ELISA assays were further tested by PCR.

DNA isolation used a DNA Purification Kit (Promega, USA) according to the manufacturer’s instructions. Briefly, 300 μL of the blood sample was placed in a sterile Eppendorf tube (1.5 mL size) and 900 μL of erythrocyte lysis solution as added. The mixture was incubated for 10 min at room temperature (22-25°C), and then centrifuged using a refrigerated Eppendorf centrifuge at 16,000 rpm for 1 min. The leukocyte pellet was dispersed by vortexing for 20 s at high speed after discarding the supernatant. Three hundred microliter of nucleic lysis solution was added to the resuspended white pellet and pipetted for 3-5 times to lyse the white blood cells. The suspension was then incubated at 37°C for 1 h, and 1.5 μL of RNase solution was added by micropipette. Incubation was continued for 15-20 min and then a 100 μL aliquot vortexed for 20 s at high speed. A small clump of protein was visible. After 4 min at room temperature, the lysate was centrifuged at 16,000 rpm. A dark brown protein pellet after centrifugation was visible. The supernatant containing total DNA was transferred to a clean 1.5 mL Eppendorf tube containing 300 μL isopropanol at room temperature. The solution was gently mixed 5-8 times by inversion until white thread-like strands of DNA formed a visible mass. The samples were then centrifuged at 16000 rpm for 5 min to recover DNA. DNA pellet was washed with 300 μL of 70% ethanol, dried, and resuspended in 60 μL of DNA rehydration solution. Extracted DNA was kept at −20°C until use in PCR analyses. Concentration and purity of DNA were confirmed spectrophotometrically. Furthermore, agarose gels with 5 μL of DNA were used to examine the quality and quantity of DNA. Finally, sheep glyceraldehyde 3-phosphate dehydrogenase was also used to confirm the quality of extracted DNA [30].

### Extraction of DNA from tissues

Briefly, 3 mL of 1× phosphate-buffered saline and 1 g of a pooled sample of tissues (uterus, testes, accessory organ, and lymph node) were added to a 10 mL tube and tissue homogenized with a tissue homogenizer. After homogenization, the suspension was centrifuged at 3000 rpm for 5 min at 4°C. Two hundred microliter of supernatants and 600 μL of nucleic lysis solution were mixed and pipetted repeatedly until no clumps of the cell were visible. The lysate was then incubated at 65°C for 15-30 min in a water bath. Three microliter of RNase was added and incubation was continued at 37°C for 15-30 min. After cooling, DNA was obtained as described above.

### Primers

**Figure-3:**
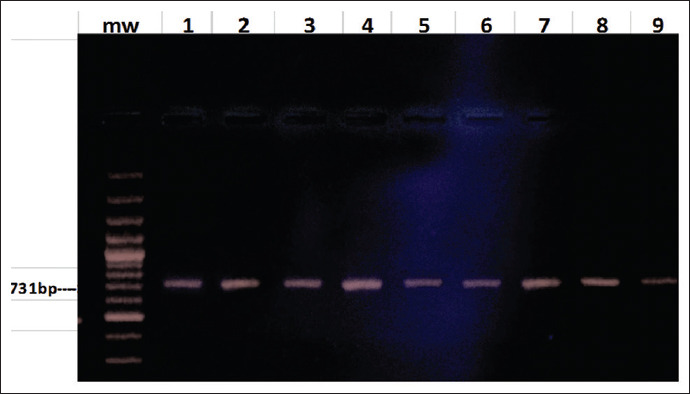
Polymerase chain reaction result in agar gel.

Primer pairs specific to the IS711 element of *B. melitensis*, 5’AAATCGCGTCCTTGCTGGTCTGA3’ and 5’TGCCGATCACTTAAGGGCCTTCAT3’, were used to confirm *B. melitensis* in tissue and blood samples. The amplified product of this primer set was 731-bp identified with agarose gel electrophoresis [30] (Figure-3).

### PCR

PCR amplification was performed using a Promega Go Taq Green Master Mix (USA). Briefly, the PCR mix included 12.5 μL of Go Taq Green Master Mix, 4.5 μL nuclease-free water, 2 μL MgCl_2_, 2 μL of every forward and reverse primers, and 2 μL of genomic DNA. A total volume of 25 μL after that was initially incubated at 94°C for 4 min. PCR was then performed with 35 cycles with 1 min for DNA denaturation at 94°C, 1 min at 50°C for primer annealing for the OMP 2 primers set, and 56°C for the *B. melitensis* specific primer set, and for polymerase-mediated primer extension at 72°C for 1.5 min. The last (45) cycle included sample incubation for 10 min at 72°C and the retained at 4°C for an unlimited time. Seven microliters of the amplified product were analyzed by electrophoresis in 1.5% agarose gels in TBE buffer with ethidium bromide. DNA bands were visualized under UV light and photographed using an AlphaImager (Alpha Innotech) image documentation system [30].

### Identification of *Brucella* species from *Brucella* isolates

Presumptive identification of *B. melitensis* was made based by observing small Gram-negative coccobacilli, and biochemical tests positive for “oxidase, catalase, and urease,” and further confirmed by antigen-antibody reaction agglutination with specific antisera (Remel Europe Ltd.). Moreover, identification, using Vitek 2 systems (version 07.01, BioMerieux), was performed with a Gram-negative bacteria colorimetric identification card (GN card) that contains different biochemical tests recommended by FAO/WHO expert committee on brucellosis [5,30].

### Statistical analysis

Statistical analyses used the Statistical Package for the Social Sciences (SPSS) for Windows® version 20.0 (SPSS Inc., Chicago, Illinois). Descriptive statistics of the variables included frequencies and prevalence.

Variables of category – *Brucella* species, territory, farm, and animal sex – were expressed in numbers and percentages. The prevalence proportion was calculated as the number of animals testing positive by RBPT, i-ELISA, or PCR, divided by the total number of animals. Correlation among factors and outcome variables were assessed using Chi-square tests. For all analyses, p<0.05 was considered significant.

## Results

### Blood test

#### RBPT

RBPT showed that 62/8500 of animals (0.729%, confidence interval [CI] 95% 0.57-0.94) were seropositive. Thirteen of 5000 (0.26%, CI 95% 0.15-0.45) and 49/3500 (1.4%, CI 95% 1.1-1.8) animals were positive in farms and quarantine, respectively. Forty-six of 6441 sheep (0.71%, CI 95% 0.54-0.95) were seropositive with 10/3420 (0.292%, CI 95% 0.16-0.54) and 36/3021 (1.192%, CI 95% 0.86-1.65) animals from farms and quarantine, respectively. Sixteen of 2059 goats (0.78%, CI 95% 0.48-1.27) were seropositive with 3/1580 (0.19%, CI 95% 0.06-0.59) and 13/479 (2.7%, CI 95% 1.6-4.6) animals farms and quarantine, respectively (Tables-4 and 5).

**Table-4:** RBPT test result.

Group	Type	Territory	RBPT results					

			Total	%	Sheep	%	Goats	%
Group 1	Unorganized farms	Northern terr.	5	0.33	3	0.38	2	0.28
		Southern terr.	3	0.25	3	0.33	0	0.00
		Moharaq terr.	0	0.00	0	0.00	0	0.00
		Middle terr.	2	0.18	1	0.15	1	0.24
	Organized farms		3	0.30	3	0.34	0	0.00
	Total (Group 1) farms		13	0.26	10	0.29	3	0.19
Group 2	Quarantine		49	1.40	36	1.19	13	2.71
Total			62	0.73	46	0.71	16	0.78

RBPT=Rose Bengal plate test

**Table-5:** RBPT result CI 95%.

	Group 1 farms	Group 2 quarantine	Total
Number of positive RBPT	13/5000	49/3500	62/8500
%	0.26	1.4	0.73
CI 95	0.15-0.45	1.1-1.8	0.57-0.94
Number of positive RBPT sheep	10/3420	36/3021	46/6441
%	0.29	1.19	0.71
CI 95	0.16-0.54	0.86-1.65	0.54-0.95
Number of positive RBPT goats	3/1580	13/479	16/2059
%	0.19	2.71	0.78
CI 95	0.06-0.59	1.6-4.6	0.48-1.27

CI=Confidence interval, RBPT=Rose Bengal plate test

#### ELISA

i-ELISA confirmed 41 of 62 RBPT-positive results, 0.482% of 8500 animals (CI 95% 0.36-0.65). Eight of 5000 (0.16%, CI 95% 0.08-0.32) and 33/3500 (0.94%, CI 95% 0.67-1.3) were confirmed positive in animals from farms and quarantine, respectively. In sheep, 30 of 6441 (0.466%, CI 95% 0.33-0.67) were positive. Of which 5/4320 (0.146%, CI 95% 0.06-0.35) and 25/3021 (0.828%, CI 95% 0.56-1.2) from farms and quarantine, respectively. Similar results for goats were 11/2059 (0.534%, CI 95% 0.3-0.96) total, 3/1580 (0.192%, CI 95% 0.06-0.59) for farms, and 8/479 (1.68%, CI 95% 0.84-3.32) for quarantine (Tables-6 and 7).

**Table-6:** i-ELISA test results.

Group	Type	Territory	i-ELISA results					

			Total	%	Sheep	%	Goats	%
Group 1	Unorganized farms	Northern terr.	3	0.20	1	0.13	2	0.28
		Southern terr.	2	0.17	2	0.22	0	0.00
		Moharaq terr.	0	0.00	0	0.00	0	0.00
		Middle terr.	2	0.18	1	0.15	1	0.24
	Organized farms		1	0.10	1	0.11	0	0.00
	Total (Group 1) farms		8	0.16	5	0.15	3	0.19
Group 2	Quarantine		33	0.94	25	0.83	8	1.67
Total			41	0.48	30	0.47	11	0.53

i-ELISA=Indirect enzyme-linked immunosorbent assay

**Table-7:** i-ELISA test result CI 95%.

	Group 1 farms	Group 2 Quarantine	Total
Number of positive i-ELISA	8/5000	33/3500	41/8500
%	0.16	0.94	0.48
CI 95	0.08-0.32	0.67-1.3	0.36-0.65
Number of positive i-ELISA Sheep	5/3420	25/3021	30/6441
%	0.15	0.83	0.47
CI 95	0.06-0.35	0.56-1.2	0.33-0.67
Number of positive i-ELISA goats	3/1580	8/479	11/2059
%	0.19	1.67	0.53
CI 95	0.06-0.59	0.84-3.32	0.3-0.96

CI=Confidence interval, i-ELISA=Indirect enzyme-linked immunosorbent assay

#### Territory distribution

For Group 1 (farms), 13/5000 (0.26%) animals were seropositive. The prevalence was 0.33%, 0.25%, 0%, 0.18%, and 0.3% for Northern, Southern, Moharaq, Middle, and organized farms, respectively. Positive results confirmed that i-ELISA was obtained for 8/5000 (0.16%) animals. The prevalence was 0.2%, 0.17%, 0%, 0.182%, and 0.1% for Northern, Southern, Moharaq, Middle, and conventional farms, respectively. For sheep, RBPT indicated that 10/3420 (0.292%) animals were seropositive. The prevalence was 0.38%, 0.33%, 0%, 0.15%, and 0.34% for Northern, Southern, Moharaq, Middle, and conventional farms, respectively. Results confirmed by i-ELISA showed that 5/3420 (0.15%) sheep were seropositive. The prevalence was 0.13%, 0.22%, 0%, 0.15%, and 0.11% for Northern, Southern, Moharaq, Middle, and conventional farms, respectively.

RBPT results showed that 3/1580 (0.19%) goats were seropositive. The prevalence was 0.28%, 0%, 0%, 0.24%, and 0% Northern, Southern, Moharaq, Middle, and conventional farms, respectively. i-ELISA confirmed all positive sera identified by RBPT.

**Figure-4:**
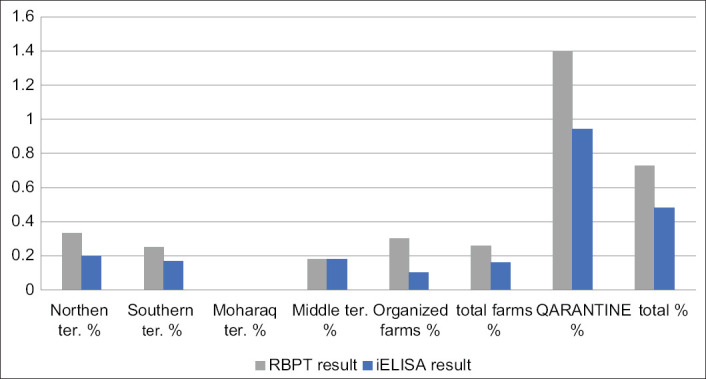
Chart of total infection rates by Rose Bengal Plate Test and indirect enzyme-linked immunosorbent assay.

**Figure-5:**
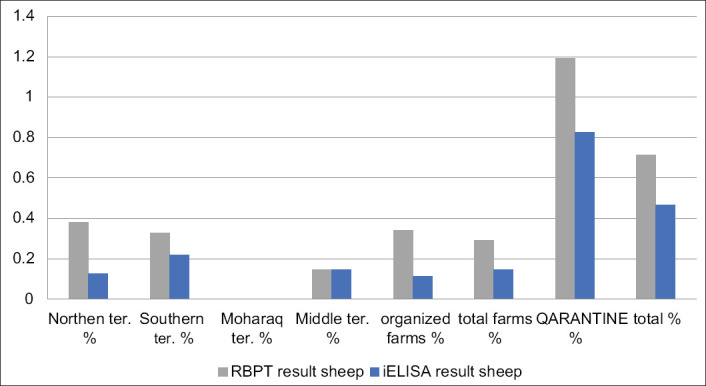
Chart of infection rates by Rose Bengal Plate Test and indirect enzyme-linked immunosorbent assay in sheep.

**Figure-6:**
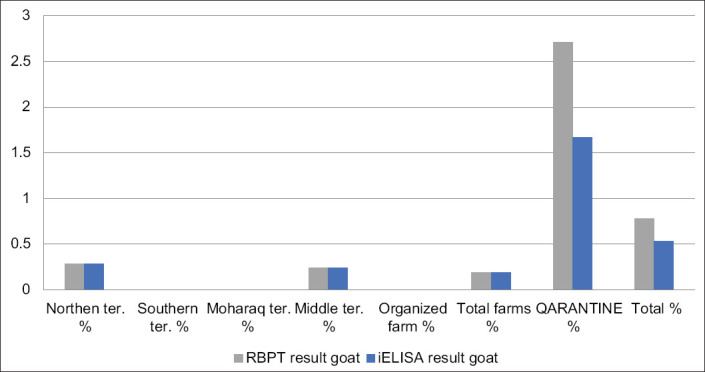
Chart showing infection rates by Rose Bengal Plate Test and indirect enzyme-linked immunosorbent assay in goats.

For Group 2 (quarantine), RBPT identified 49/3500 (1.4%) animals and 1.19% and 2.71% for sheep and goats, respectively, as seropositive. For i-ELISA, 33/3500 (0.94%) and 0.83% and 1.67% for sheep and goats, respectively, were confirmed (Tables-4 and 6, Figures-4-6)

### Farm view rates

Examining data by showed 5/259 (1.93%) of farms were seropositive by RBPT for one or more animals. Two, 1, 0, 1, and 1 farms show the prevalence *Brucella* of 2.35%, 1.39%, 0%, 1.61%, and 12.5% in Northern, Southern, Moharaq, Middle, and conventional farms, respectively. i-ELISA confirmed that 4/259 (1.54%) of farms were seropositive. The one farm that was not confirmed was in the Northern Territory (Table-8). RBPT analyses for sheep farms showed that 4/245 (1.68%) farms were seropositive. One, 1, 0, 1, and 1 farms show the prevalence of 1.35%, 1.45%, 0%, 1.33%, and 12.5% in Northern, Southern, Moharaq, Middle, and conventional farms. i-ELISA confirmed all RBPT results (Table-9). RBPT analyses found that 2/128 (1.56%) farms were seropositive. One, 0, 0, 1, and 0 farms show the prevalence of 1.92%, 0%, 0%, 2.56%, and 0% in Northern, Southern, Moharaq, Middle, and conventional farms, respectively. The same result was obtained when confirmed by i-ELISA (Table-10).

**Table-8:** Farm rate tests results.

Group	Type	Territory	Sample		RBPT			i-ELISA		
		
			Number of samples	Number of farms	Result	Number of positive farms	Percentage of positive farms	Result	Number of positive farms	Percentage of positive farms
Group 1	Unorganized farms	Northern terr.	1500	85	5	2	2.35	3	1	1.18
		Southern terr.	1200	72	3	1	1.39	2	1	1.39
		Moharaq terr.	200	32	0	0	0.00	0	0	0.00
		Middle terr.	1100	62	2	1	1.61	2	1	1.61
	Organized farms		1000	8	3	1	12.50	1	1	12.50
	Total (Group 1) farms		5000	259	13	5	1.93	8	4	1.54

RBPT=Rose Bengal plate test, i-ELISA=Indirect enzyme-linked immunosorbent assay

**Table-9:** Sheep in farms rate results.

Group	Type	Territory	Sample		RBPT			i-ELISA		
		
			Number of samples	Number of farms	Result	Number of positive farms	Percentage of positive farms	Result	Number of positive farms	Percentage of positive farms
Group 1	Unorganized farms	Nortdern terr.	789	74	3	1	1.35	1	1	1.35
		Soutdern terr.	912	69	3	1	1.45	2	1	1.45
		Moharaq terr.	148	19	0	0	0.00	0	0	0.00
		Middle terr.	688	75	1	1	1.33	1	1	1.33
	Organized farms		883	8	3	1	12.50	1	1	12.50
	Total (Group 1) farms		3420	245	10	4	1.68	5	4	1.68

RBPT=Rose Bengal plate test, i-ELISA=Indirect enzyme-linked immunosorbent assay

**Table-10:** Goats in farms rate results.

Group	Type	Territory	Sample		RBPT			i-ELISA		
		
			Number of samples	Number of farms	Result	Number of positive farms	Percentage of positive farms	Result	Number of positive farms	Percentage of positive farms
Group 1	unorganized farms	Nortdern terr.	711	52	2	1	1.92	2	1	1.92
		Soutdern terr.	288	27	0	0	0.00	0	0	0.00
		Moharaq terr.	52	5	0	0	0.00	0	0	0.00
		Middle terr.	412	39	1	1	2.56	1	1	2.56
	Organized farms		117	5	0	0	0.00	0	0	0.00
	Total (Group 1) farms		1580	128	3	2	1.56	3	2	1.56

RBPT=Rose Bengal plate test, i-ELISA=Indirect enzyme-linked immunosorbent assay

### Sex-related rate

RBPT analyses found that 1/423 (0.24%) males were seropositive with the single positive result from an animal in the Northern Territory. In contrast, 12/4577 (0.26%) female animals were seropositive. The prevalence was 0.3%, 0.28%, 0%, 0.2%, and 0.31% in Northern, Southern, Moharaq, Middle, and conventional farms, respectively.

i-ELISA did not confirm the single seropositive result in the male animals. Eight of 4577 (0.16%) females were seropositive in i-ELISA. The prevalence was 0.22%, 0.19%, 0%, 0.2%, and 0.1% in Northern, Southern, Moharaq, Middle, and conventional farms, respectively.

**Figure-7:**
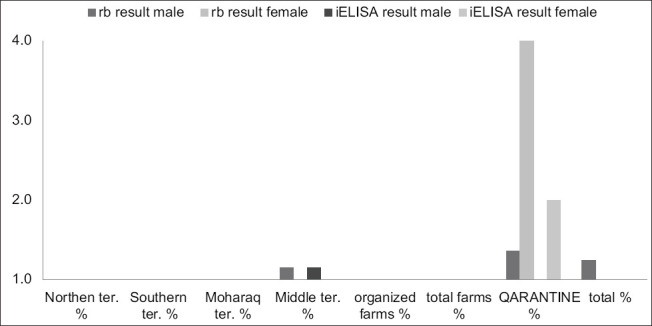
Chart showing male and female rate.

For Group 2 (quarantine), 47/3450 (1.36%) and 32/3450 (0.93%) male animals were identified by RBPT and i-ELISA, respectively, and were 2/50 (4.0%) and 1/50 (2.0%) female animals using RBPT and i-ELISA, respectively (Table-11 and Figure-7).

**Table-11:** Male and female rate.

Group	Type	Territory	Sample			RBPT			i-ELISA		

			Number of samples	Number of sample male	Number of sample female	RBPT result	RBPT result male	RBPT result female	i-ELISA result	i-ELISA result male	i-ELISA result female
Group 1	Unorganized farms	Northern terr.	1500	150	1350	5	1	4	3	0	3
		Northern terr. %				0.33	0.67	0.30	0.20	0.00	0.22
		Southern terr.	1200	123	1077	3	0	3	2	0	2
		Southern ter. %				0.25	0.00	0.28	0.17	0.00	0.19
		Moharaq terr.	200	38	162	0	0	0	0	0	0
		Moharaq terr. %				0.00	0.00	0.00	0.00	0.00	0.00
		Middle terr.	1100	87	1013	2	0	2	2	0	2
		Middle terr. %				0.18	0.00	0.20	0.18	0.00	0.20
	Organized farms		1000	25	975	3	0	3	1	0	1
					0.30	0.00	0.31	0.10	0.00	0.10	
	Total farms		5000	423	4577	13	1	12	8	0	8
	Total farms %					0.26	0.24	0.26	0.16	0.00	0.17
Group 2	Quarantine		3500	3450	50	49	47	2	33	32	1
	Quarantine %					1.40	1.36	4.00	0.94	0.93	2.00
Total			8500	3873	4627	62	48	14	41	32	9
Total %						0.73	1.24	0.30	0.48	0.83	0.19

RBPT=Rose Bengal plate test, i-ELISA=Indirect enzyme-linked immunosorbent assay

## PCR results

PCR confirmed brucellosis in blood samples in 41 samples that were positive in RBPT and i-ELISA analyses. PCR confirmed 18/8500 samples (0.21%, CI 95% 0.13-0.34). Three of 5000 (CI 95%, 0.019-0.19) and 15/3500 (0.43%, CI 95% 0.26-0.71) were confirmed animals from Group 1 (farms) and Group 2 (quarantine), respectively. Fifteen of 6441 (0.466%) samples from sheep were confirmed with 2/4320 (0.06%) and 13/3021 (0.43%) in animals from farms and quarantine, respectively. Confirmation results for goats were 3/2059 (0.146%), 1/1580 (0.06%), and 2/479 (0.42%) total, farm, and quarantine animals, respectively (Tables-12 and 13).

**Table-12:** PCR results blood samples.

Group	Type	Area	PCR serum					

			Total	%	Sheep	%	Goats	%
Group 1	Unorganized farms	Northern terr.	1	0.07	0	0.00	1	0.14
		Southern terr.	1	0.08	1	0.11	0	0
		Moharaq terr.	0	0	0	0	0	0
		Middle terr.	1	0.09	1	0.15	0	0
	Organized farms		0	0	0	0	0	0
	Total (Group 1) farms		3	0.06	2	0.06	1	0.06
Group 2	Quarantine		15	0.43	13	0.43	2	0.42
Total			18	0.21	15	0.23	3	0.15

PCR=Polymerase chain reaction

**Table-13:** PCR results blood samples CI 95%.

Group	PCR serum					

	Total	%	Sheep	%	Goats	%
Group 1 farms	3/5000	0.06	2	0.06	1	0.06
	CI 95	0.019 - 0.19				
Group 2 Quarantine	15/3500	0.43	13	0.43	2	0.42
	CI 95	0.26-0.71				
Total	18/8500	0.21	15	0.23	3	0.15
	CI 95	0.13-0.34				

PCR=Polymerase chain reaction, CI=Confidence interval

### Tests on tissue samples

#### Brucella isolation and identification

*Brucella* was isolated and identified for brucellosis in tissue samples from 14 to 30 culled or slaughtered animals that were seropositive by RBPT and i-ELISA. All *B. melitensis* isolates were Biovar 1 and were less prevalent in farm animals (3/5000, 0.06%) than quarantined animals (11/3500, 0.31%). Eleven of 6441 sheep (0.18%) were confirmed positive, 2/4320 (0.07%) and 9/3021 (0.3%) from Group 1 (farms) and Group 2 (quarantine), respectively. The results for goats were 3/2059 (0.16%), 1/1580 (0.072%), and 2/479 (0.42%) for total, farm, and quarantined animals, respectively (Table-14).

**Table-14:** Results of tissue samples examination.

Group	Type	Territory	Media culture			PCR t. sample		
	
			Total	Sheep	Goats	Total	Sheep	Goats
Group 1	Unorganized farms	Northern terr.	2	1	1	1	1	0
		Northern terr. %	0.13	0.13	0.14	0.07	0.13	0
		Southern terr.	1	1	0	1	1	0
		Southern terr. %	0.08	0.11	0	0.08	0.11	0
		Moharaq terr.	0	0	0	0	0	0
		Moharaq terr. %	0	0	0	0	0	0
		Middle terr.	0	0	0	1	0	1
		Middle terr. %	0	0	0	0.09	0	0.24
	Organized farms		0	0	0	1	1	0
	Organized farms %			0	0	0.10	0.11	0
	Total farms		3	2	1	4	3	1
	Total farms %		0.06	0.07	0.07	0.08	0.09	0.08
Group 2	Quarantine		11	9	2	19	15	4
	Quarantine %		0.31	0.30	0.42	0.54	0.50	0.84
Total			14	11	3	23	18	5
Total %			0.17	0.18	0.16	0.27	0.28	0.26

PCR=Polymerase chain reaction

#### PCR for tissue samples

PCR confirmed *B. melitensis* in 23/30 tissue samples (0.27%) with 4/5000 (0.08%) and 19/3500 (0.54%) in animals from Group 1 (farms) and Group 2 (quarantine), respectively (Table-14). Eighteen of 6441 (0.28%) sheep were confirmed with 3/4320 (0.09%) and 15/3021 (0.5%) in sheep from Group 1 (farms) and Group 2 (quarantine), respectively. The results were 5/2059 (0.26%) for goats with 1/1580 (0.08%) and 4/479 (0.84%) in animals from farms and quarantine (Table-14).

## Discussion

Brucellosis is an important zoonosis that causes abortion in naturally infected small ruminants and is of great public health concern in many countries [30,31]. The results of the study were part of an effort to develop a method for rapid and accurate brucellosis diagnosis. Such a method is critical to support effective eradication and monitoring programs.

Many factors that affect brucellosis seroprevalence in small ruminants could be associated with frequent introduction of purchased animals into flocks, including the absence of quarantine/segregation, mixing of different species of infected flocks, improper safe hygienic disposal of aborted fetuses placental membranes, contact of healthy animals with contaminated drinking water, grassing yards and feed, and lack of vaccination and control strategies for small ruminants [30-32].

Brucellosis control and eradication from small ruminants (sheep and goats) require an appropriate serological method for brucellosis diagnosis in the infected or endemic areas. Diagnostic tests used may not reveal all animals infected (false-negatives) or may show infection when it not present (false-positives) due to a long period of incubation, latency, or criteria used to interpret the results [31,32]. Isolation and identification of organisms are the diagnosis gold standard, but it is cumbersome, takes several days to weeks, and poses a higher risk to laboratory personnel. Hence, the diagnosis of brucellosis largely depends on the use of two or more tests to confirm infection [32]. RBPT is a current screening test, and i-ELISA is used as a confirmatory test used for *B. melitensis* infection in small ruminants (sheep and goats) [31-36]. In comparison to i-ELISA, Sn, and Sp of the RBPT are lower. However, RBPT is convenient because of low cost, feasibility, and reliability as a field diagnostic test compared with i-ELISA [37,38]. The latter technique provides acceptable sensitivity and specificity [39,40].

The overall brucellosis prevalence seropositivity in the study area was 0.48% by RBT and i-ELISA, which is lower than that found in Al-Ahsa KSA which was 1.1% [41], Alkamil, KSA 5.88% [10], Abu Dhabi Emirate 8.3% [42], Oman 2.4%, (Dhofar) 8.6%, Northern governorates 0.97%, Al Aqaieb 24%, and Al Helailat 40%. While in Al Ghilayil, Al Qasha’e, Da’anAlhamra, Al Sarah, Hail Al Hedap, and Shnoot are free from brucellosis [13,43].

*Brucella* infection seroprevalence in small ruminants was determined based on screening with RBPT and confirmation by i-ELISA. Twenty-one samples tested positive by RBPT but negative by i-ELISA. The higher sensitivity of i-ELISA due to its recognition of cytosolic antigen S-LPS fragments may decrease cross-reaction with other Gram-negative bacteria [5,44,45]. Other bacteria share similar epitopes with *Brucella* [5].

Secondary can refer acidified antigens in RPBT usually produce prozoning phenomena (false positive result), [44]. Hence, i- ELISA has been evaluated for many years for their better sensitivity to detect anti-*Brucella* antibodies in all species especially small ruminant, several studies reported that i-ELISA is more sensitive than conventional tests [13] assessed the indirect ELISA efficacy in comparison with CFT and RBPT on sera from *B. melitensis* infected ewes. Indirect ELISA can be a useful screening tool and could be used alone or in addition to RBT. The present i-ELISA performance is consistent with the study of Nielsen *et al*. [46]. The performance evaluation of infected and vaccinated sheep and goats showed that i-ELISA outperformed other tests and recommended it for diagnosis of Ovine-Caprine brucellosis. However, low performance with vaccinated animals limits i-ELISA to instances where animals have no history of vaccination to avoid false-positive results [11,47].

Estimated infection rates in sheep were 0.71% and 0.47% by RBT and i-ELISA, respectively, and in goats, 0.78% and 0.53% by RBT and i-ELISA, respectively. No significant difference existed between these species. Furthermore, a significant difference was observed between species in Group 2 (quarantine). Results for goats (2.7%) and (1.7%) were higher than for sheep (1.2) and (0.8%) by RBT and i-ELISA, respectively. This result may reflect infection rates in the country of origin and local control programs. Sheep and goats were imported from different countries. In contrast, a higher prevalence of sheep has also been reported [38,41,43].

Sheep behavior may also be a factor since they tend to gather in parturition and at night, which increases the potential for disease transmission. Goats do not display this behavior [48]. Larger herds of sheep may be more likely to show at least one positive case than small herds and are typically associated with mass livestock management. Infection in these herds may spread due to closer contact among animals and their *Brucella*-containing excretions [49].

Seroprevalence was not significantly different between males and females in Group 1 (farms) but showed a higher incidence in males in Group 2 (quarantine). This result contrasts with other reports [6,50,51]. Statistical analysis shows that males and females are equally susceptible to *Brucella* infection. Thus, the present results may be affected by the small number of males examined from farms and the few females from quarantine.

Serological testing with RBT and i-ELISA for *Brucella* infections is widely accepted. However, serum agglutination tests (and to a lesser extent RBPT) are less suitable for diagnosis of chronic brucellosis since they mainly depending on IgM and IgG detection. IgM found in sera in chronic infection will decline and become undetectable [50]. ELISA remains a useful epidemiological tool that provides for investigation of the infective status of herds [5,51-56].

Information on *Brucella* prevalence in small ruminants (goats and sheep) is critical to preventive control measures for brucellosis. However, seroprevalence information for brucellosis in the gulf province has been scant, though small ruminants are major livestock and traditionally the main sources of meat. Infection is often under-detected in this region despite inflicting a high economic and health burden, mainly due to a lack of concern at the individual level in rural areas. Moreover, the present study results are useful for policymakers to evaluate the status of the disease in livestock. Finally, the study provides baseline data for further study of *Brucella* infections and for planning control and eradication strategies.

PCR and other genetic techniques are broadly used for the rapid detection of brucellosis [57] and could be considered in future studies in the gulf. PCR confirmed brucellosis in blood samples in 18 of 41 samples that were seropositive by RBPT and i-ELISA. Of these samples, 3 and 15 were confirmed positive in animals from farms and quarantine, respectively. Fifteen sheep were confirmed positive, 2 and 13 in animals from farms and quarantine, respectively. Three samples from goats 3/2059 (0.146%) were confirmed positive, with one from farms. The results indicate that diagnosis of brucellosis by PCR using blood as a sample is applicable but not simple. In the present investigation, further effort was made for diagnosing brucellosis from blood samples [22,57].

Only 18 samples were positive by PCR compared to 62 by RBPT and 41 by i-ELISA. Wide variation in samples detected could be due to many factors. PCR detects DNA, which may be in low quantity in blood samples even though antibody titer is quite high. Alternatively, titer in serum may be below detectable levels, but the amount of DNA may be sufficient for detection by PCR. PCR may detect as little as five fg of DNA [58]. Furthermore, standardized conditions for RBPT antigen suitable for diagnosing bovine infection are not adequate for sheep and accounts for the low sensitivity of RBPT in small ruminants [59]. This report is preliminary and indicates that PCR can also be applied for the diagnosis of brucellosis in animals, using blood samples, for more rapid and accurate brucellosis diagnosis.

From 30 tissue samples collected from culled or slaughtered animals that were seropositive, only 23 samples were detected as positive by PCR. Thus, PCR did not identify seven seropositive samples as positive. Isolation identified only 14 samples of *B. melitensis*, and confirmed Biovar 1. No bacteria could be isolated from 16 seropositive samples. Isolation and cultivation are a gold standard diagnostic technique for brucellosis since it is specific and permits biotyping to aide epidemiological analysis [60]. Only the organism can provide a definitive profile and firm confirmation of infection. However, negative result does not exclude the presence of brucellosis [61]. Typing of *Brucella* was performed for strains isolated from tissue samples and lymph nodes from slaughtered animals verified to be serologically positive. All isolates were typical of *B. melitensis* and were identified as *Br. melitensis* Biovar 1. These results are consistent with the previous reports [61-64]. The primary hosts for *B. melitensis* are small ruminants, suggesting that the sensitivity of serological tests is higher than that of the culture method. A similar conclusion was reached by Sayour [62], who concluded that the best specific diagnostic assay is the isolation of the causative organism; however, this method suffers the disadvantages of low sensitivity and a requirement for extended time for tissue preparation and culture.

Some research indicates that PCR-ELISA is more sensitive than other molecular methods. This method is more effective and accurate than PCR, serology, and culture of bacteria [65]. PCR is more sensitive and easily applicable than bacterial culture [6,11]. Further, some studies indicate a real transmission risk to both butchery personnel and consumers. Accurate and sensitive testing of animals before slaughter and marketing is needed to prevent the spread of human infection [66].

## Conclusion

The overall incidence of brucellosis is 0.48%, and 0.16% in farms. In this area of the gulf, the disease seems to be under control, and a continuous effort is needed to maintain control and eventually completely eradicate brucellosis. Additional support is needed for testing and slaughterhouse monitoring. In quarantined (imported) animals, brucellosis infection in the slaughterhouse (0.94%) could pose a risk for transmission and spread of infection. Effort is needed to monitor this threat, and PCR is a sensitive and time-saving test for brucellosis diagnosis. All 14 confirmed positive samples were Biovar 1 dominant.

## Authors’ Contributions

ME, AE, and FS planned the study. ME collected the samples, performed the experiments, processed the experimental data, performed the analysis, drafted the manuscript, and designed the figures and performed the calculations. AE and FS supervised the findings of this work. All authors discussed the results and contributed to the final manuscript.

## Competing Interests

The authors declare that they have no competing interests.

## Publisher’s Note

Veterinary World remains neutral with regard to jurisdictional claims in published institutional affiliation.
